# Regulation of natural killer cell activity by glucocorticoids, serotonin, dopamine, and epinephrine

**DOI:** 10.1038/s41423-020-0477-9

**Published:** 2020-06-05

**Authors:** Silvia Capellino, Maren Claus, Carsten Watzl

**Affiliations:** Department for Immunology, Leibniz Research Centre for Working Environment and Human Factors (IfADo) at TU Dortmund, Dortmund, Germany

**Keywords:** Natural Killer Cells, Catecholamines, Glucocorticoids, Neurotransmitters, Innate lymphoid cells, Immunosuppression, Chronic inflammation

## Abstract

The immune system and the nervous system are highly complex organs composed of various different cells that must interact with each other for proper function of the system. This communication can be mediated by soluble factors. The factors released by the nervous system (neurotransmitters) differ from those released by the immune system (cytokines). Nevertheless, the nervous and immune systems can influence each other’s activity because immune cells express neurotransmitter receptors, and neurons express cytokine receptors. Moreover, immune cells can synthesize and release neurotransmitters themselves, thus using neurotransmitter-mediated pathways via autocrine and paracrine mechanisms. Natural killer (NK) cells are innate lymphocytes that are important for early and effective immune reactions against infections and cancer. Many studies have shown the strong influence of stress and the nervous system on NK cell activity. This phenomenon may be one reason why chronic stress leads to a higher incidence of infections and cancer. Here, we review the effects of neuroendocrine factors on the different activities of NK cells. Understanding the effects of neuroendocrine factors on NK cell activities during physiological and pathophysiological conditions may result in novel therapeutic strategies to enhance NK cell functions against tumors.

## Introduction

Both the immune system and the nervous system are highly complex organs that have some interesting similarities. Both organs are composed of various different cells that must interact with each other for proper function of the system. For this interaction, cellular communication is key. This communication is mediated by direct cellular contacts (e.g., synapse formation between neurons or between immune cells) and by soluble mediators (neurotransmitters or cytokines). Interestingly, communication is not limited to cells of each system. Many examples have shown that the nervous system and the immune system interact and thereby influence each other’s activity. For example, during inflammatory responses of the immune system against infections, the cytokines produced by immune cells can also affect cells of the nervous system and mediate what is called “sickness behavior”.^1^ Communication between the immune system and the nervous system is bidirectional. In this review, we will focus on how the nervous system influences the activity of the immune system using natural killer (NK) cells as an example.

## The nervous system and its neurotransmitters

**Fig. 1:**
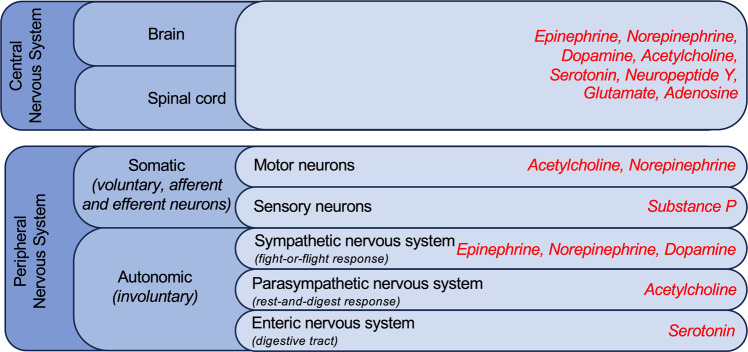
Diagram showing the major divisions of the human nervous system. The released neurotransmitters are shown in red

The nervous system is responsible for coordination, movements, thoughts, and processing, and it is divided into the central and peripheral nervous systems. The central nervous system consists of the brain and spinal cord, and is responsible for integrating and coordinating the activities of the entire body. Through these physical structures, thought, emotion, and sensation are experienced, and body movements are coordinated. The peripheral nervous system consists of all neurons that exist outside of the brain and spinal cord, and connects the central nervous system to various parts of the body. This system includes long nerve fibers as well as ganglia. Depending on the function, this system is divided into the autonomous nervous system, responsible for involuntary function, and the somatic nervous system, which regulates voluntary movements and includes afferent neurons (Fig. 1).

For nerve-to-nerve communication, some neurons communicate via electrical synapses through the use of gap junctions, but most neurons synthesize and release neurotransmitters. There are a large number of neurotransmitters in the human body, varying from very small purines (adenosine, ATP) to polypeptides such as somatostatin. Neurotransmitters are normally released in the synaptic cleft and bind to postsynaptic neurons or undergo reuptake into the presynaptic neuron. However, they can also diffuse in the blood and bind to nonneuronal cells, or they can be released from efferent nerve endings directly in peripheral organs, such as the spleen, lymph nodes, glands, the intestine, and other organs.

Catecholamines (adrenaline, noradrenaline, and dopamine), neurotransmitters of the sympathetic nervous system, and acetylcholine, neurotransmitters of the parasympathetic nervous system, are released in many peripheral organs and directly act on the body to control the fight-or-flight response (sympathetic nervous system) and the rest-and-digest response (parasympathetic nervous system).^2^ The amount of dopamine in the peripheral organs has been summarized in a recent review,^3^ which reported physiologically active concentrations of dopamine in the colon, heart, lungs, blood, and many other organs. Similarly, the peripheral concentrations of all three catecholamines and their effects on peripheral organs and tissues, as well as on memory in the brain, have been reviewed,^2^ thus highlighting the complex and important effect of the sympathetic nervous system on body functions. In addition, acetylcholine has peripheral effects on endothelial cells, lymphoid organs, and other nonneuronal cells, despite the anatomical distance from cholinergic nerves and the presence of degrading enzymes in the blood. One possible explanation for the distant action of acetylcholine is the presence of a high concentration of the acetylcholine-synthesizing enzyme in human plasma.^4^

In addition, neurotransmitters of the central nervous system, such as glutamate,^5^ are detected in the peripheral organs without any evidence of peripheral innervation. This phenomenon is because peripheral, nonneuronal cells can also synthesize and release neurotransmitters and use them in a paracrine or autocrine manner. For example, immune cells and other nonneuronal cells were shown to synthesize catecholamines in physiologic^6–10^ as well as in pathologic conditions,^11–14^ suggesting that neurotransmitters may have crucial modulatory effects on these cells. Notably, cholinergic effects in the spleen were shown to be due to sympathetic activation of T cells, which then produce and release acetylcholine.^15^ Acetylcholine synthesis was also demonstrated in murine NK cells, with upregulation of the NK cell cholinergic system during autoimmune activation.^16^ In addition, many other neurotransmitters can be produced and released outside of the nervous system,^17,18^ suggesting a crucial, so far underestimated role for neurotransmitters in physiology and the immune response.

## NK cells

NK cells are innate lymphocytes that are important for early and effective immune reactions against infections and cancer.^19^ To mediate these important responses, NK cells have essentially two effector mechanisms—cellular cytotoxicity and the production of cytokines. These activities can be stimulated by different surface receptors.^20^ Via cytokine receptors, NK cells can respond to cytokines that are produced during the early phases of an infection, such as type I interferon, IL-12, and IL-18. This phenomenon results in the activation of NK cells and the production of IFNγ and other cytokines, which initiate and shape the following adaptive immune response. Via a variety of activating and inhibitory surface receptors,^21^ NK cells can interact with infected or transformed cells and mediate cellular cytotoxicity. For this activity, close contact between the two cells is necessary, which is often referred to as the immunological synapse.^22^ To form this contact, adhesion receptors such as integrins are essential. Within this synapse, activating and inhibitory NK cell receptors can interact with their respective ligands on the target cell. The integration of their positive and negative signals inside NK cells ultimately determines whether NK cells are activated.^23^ Upon NK cell activation, the synapse is stabilized, and intracellular vesicles containing cytotoxic molecules, such as perforin and granzymes, are polarized toward the contact site.^24^ The content of these vesicles, which are also called granules, is then released into the synaptic cleft, resulting in the death of the attached target cell.^25^ As an alternative mechanism, NK cells can express ligands for death receptors such as TRAIL or FasL on their surface, which upon interaction with their respective receptors, can also induce target cell apoptosis. Both killing mechanisms seem to be differentially used during the serial killing activity of NK cells, by which they can eliminate several target cells in a serial fashion.^26^

Through their cytokine production and via their cellular cytotoxicity, NK cells can contribute to effective immune reactions against viral infections and transformed cells. NK cell responses against cytomegalovirus (CMV) infections have been very well characterized, as NK cells seem to be particularly well equipped to react against this virus via specific receptors.^27^ In addition, NK cells are important for immunosurveillance against tumors, and NK cell responses against hematological malignancies have been well studied. These analyses have led to the use of NK cells in several immunotherapeutic approaches against leukemia and other forms of cancer.^28,29^

NK cells are part of a larger group of innate lymphocytes, commonly referred to as innate lymphoid cells (ILCs).^30^ While NK cells are cytotoxic effector cells, other ILCs, such as the subgroups ILC1, ILC2, and ILC3, have a helper function by producing different cytokines. These helper subsets are mostly found in tissues, especially at mucosal sites, where they can rapidly react to infections. Given their location, ILCs have been shown to be influenced by several different soluble mediators of the nervous system, which are directly released into the tissue by neurons. As the regulation of helper ILCs by neurotransmitters and neuropeptides has been the subject of several recent reviews,^31^ we will focus here on the regulation of NK cell activities by the nervous system.

## Regulation of NK cells by glucocorticoids

**Fig. 2:**
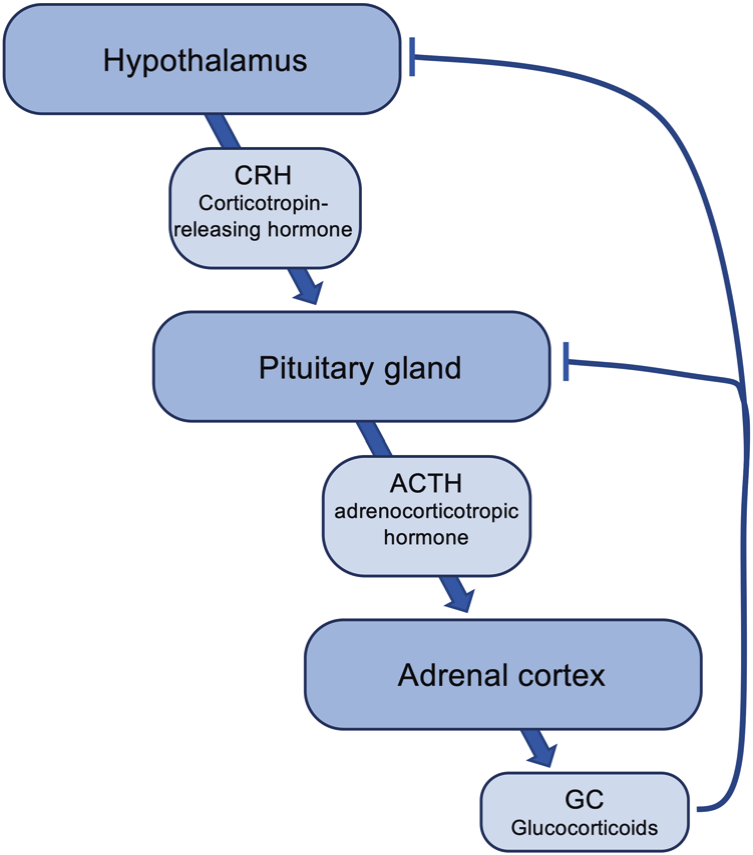
Regulation of the hypothalamic–pituitary–adrenal (HPA) axis. See text for details

Glucocorticoids (GCs) are steroid hormones that are released during the so-called stress response by activation of the hypothalamic–pituitary–adrenal (HPA) axis (Fig. 2). During this response, the hypothalamus is activated, resulting in the release of corticotropin-releasing hormone (CRH). This change stimulates the secretion of adrenocorticotropic hormone (ACTH) by the pituitary gland of the anterior lobe. Upon ACTH stimulation, the adrenal cortex then releases GCs into the bloodstream. While this has a negative feedback effect on the hypothalamus and the pituitary gland to inhibit the production of CRH and ACTH, GCs regulate a wide variety of physiological processes, including metabolism, circadian rhythm, and immunity. GCs mediate their effects by binding to GC receptors, which are ubiquitously expressed by almost all cells. In general, the effect of GCs on the immune system is considered to be anti-inflammatory by inhibiting the production of proinflammatory cytokines, such as IL-6, TNF, IL-1ß, or IL-12 by monocytes, macrophages, and dendritic cells.^32^ Given this anti-inflammatory function, GCs are also widely used as therapeutics to treat inflammatory disorders.

**Fig. 3:**
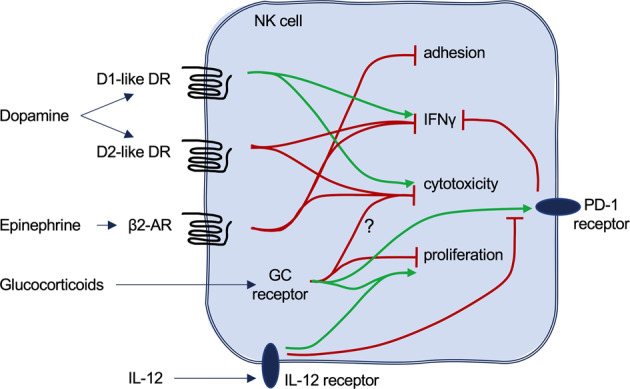
Effects of dopamine, epinephrine, and glucocorticoids on NK cell activities. Green arrows symbolize activation events, and red lines symbolize inhibition. See text for details

Approximately 40 years ago, GCs were shown to have an inhibitory effect on NK cell functions.^33–35^ This finding has been confirmed by many labs, and more details about the effect of GCs on NK cell reactivity have been reported.^34,36–41^ Through binding to GC receptors, GCs typically alter gene transcription. This change results in reduced expression of several genes that are important for NK cell functions.^37,39^ Via reduced expression of the integrin LFA-1, adhesion to target cells is reduced by GCs.^38,41^ In addition, effector molecules, such as perforin, granzyme B, and granzyme A, are inhibited in their expression, resulting in reduced cytotoxic activity of NK cells.^38,41^ More importantly, the production of IFNγ, as a key NK cell cytokine, is inhibited by GCs.^42,43^ These findings indicate a general inhibition of NK cell activities by GCs (Fig. 3). Given that GCs are secreted during the stress response, this phenomenon could provide an important link between (chronic) stress and the suppression of immune functions, leading to increased susceptibility to infections and reduced immunosurveillance of tumors under such conditions.

The inhibitory effect of GCs on NK cells is relevant for regulating immune responses. In mice with an NK cell-specific deletion of the GC receptor, CMV infections could no longer be controlled, leading to reduced survival of the deficient animals upon CMV infection.^44^ This result was caused by excessive IFNγ production by NK cells in the spleen of GC receptor-deficient mice. Mechanistically, infection by CMV activates the HPA axis, resulting in the release of GCs. These factors induce the expression of the inhibitory receptor PD-1 on NK cells in the spleen, which regulates and limits their IFNγ production (Fig. 3). Without this regulation, excessive IFNγ production by NK cells causes immune-mediated pathology, leading to the increased mortality of the GC receptor-deficient mice during infection. This result demonstrates that the GC-mediated inhibition of NK cell functions is important for preventing excess immune reactions and inflammatory disorders.

While the inhibitory effect of GCs on the production of cytokines such as IFNγ by NK cells is well established, there are conflicting reports about the regulation of other NK cell activities. Treatment of NK cells with GCs can result in reduced cytotoxic activity as described above. However, in the absence of GC-receptor expression, viral clearance after CMV infection was not altered compared with that in wt mice.^44^ As viral clearance is dependent on NK cell cytotoxicity, this finding would indicate that GCs produced during CMV infection do not affect NK cell cytotoxicity while still inhibiting IFNγ production. It could be that different time frames or concentrations are necessary for GCs to affect the two different NK cell activities. In addition, the location of NK cells in different organs may be important. In the CMV infection model, GCs upregulated PD-1 only on NK cells in the spleen but not in the liver, which was suggested to be linked to an IL-12-mediated inhibition of this GC effect.^44^ Interestingly, IL-12 was also reported to affect other functions of GCs. The proliferation of NK cells is typically inhibited by GCs. In contrast, GCs were found to enhance the proliferation of human NK cells upon exposure to IL-2 and IL-12.^43^ Therefore, IL-12 signaling may block or even reverse the effect of GCs on NK cells. This interesting link between IL-12, which is produced during infections, and GCs in NK cells, will need to be investigated further (Fig. 3).

## Regulation of NK cells by monoamines

Monoamines are a group of important bioactive substances of the CNS that are considered to act as neuromodulators and regulate important functions, such as motor control, cognition, emotion, and memory processing. The major monoamines are serotonin, dopamine, and noradrenaline. Interestingly, all these substances are known to influence NK cell activities outside of the CNS.

### Serotonin

Peripheral serotonin (5-hydroxytryptamine, 5-HT) is a derivative of tryptophan and is mainly produced by gut chromaffin cells. Serotonin is taken up from the bloodstream by platelets and released upon activation.^45^ Based on sequence homology, 7 classes of serotonin receptors (5-HTR) are known and comprise several subclasses. Six of these 5-HTRs are members of the G protein-coupled receptor superfamily and signal through different heterotrimeric G proteins: 5-HT_1_Rs inhibit cAMP production through Gαi/0, while 5-HT_4_Rs, 5-HT_6_Rs, and 5-HT_7_Rs couple to Gαs, stimulate adenylate cyclase, and increase cAMP production. The 5-HT_2_Rs activate the PLC/IP3/Ca^2+^ pathway through Gαq/11, and 5-HT3R belongs to the four-transmembrane-domain ligand-gated cation channel receptor superfamily and stimulates NO synthase and cGMP production (reviewed in ref. ^46^). However, knowledge about the expression of 5-HTRs on NK cells is very limited. The first connection between serotonin and NK cell function was found in the early 1990s, when Hellstrand et al. identified indirect effects of serotonin on NK cells through the regulation of monocytes by serotonin. Monocytes can inhibit basal and stimulated NK cell cytotoxicity, as well as cytokine production in a cell contact-dependent manner. This inhibition was abrogated by the addition of serotonin.^47,48^

Evaluation of lymphocyte subsets in patients suffering from major depressive disorder who were treated with selective serotonin reuptake inhibitors (SSRIs) led to varying results. A short-term 4-week treatment with SSRIs enhanced NK cell cytotoxicity but not NK cell numbers,^49,50^ whereas long-term treatment led to increased NK cell counts.^51^ However, no evidence for direct effects of SSRIs on NK cells was found. A flow cytometry-based study on Alzheimer’s patients suggests that NK cells may express serotonin receptors. However, the percentages reported in this study are extremely low, raising the question of functional significance.^52^ Recently, a systematic screen of commonly used drugs on isolated NK cells supported a direct effect of serotonin on NK cells. The serotonin receptor agonist quipazine was found to enhance NK cell function, whereas various dopamine/serotonin antagonists inhibited CD16-mediated NK cell function.^53^ Moreover, serotonin was shown to enhance the migratory potential, but not the effector functions of the KHYG-1 NK cell line through 5-HT_2A_R and 5-HT_2B_R.^54^ Thus, serotonin seems to modulate NK cell function in direct and indirect ways.

### Dopamine

Dopamine is a neurotransmitter of the central nervous system controlling movement, emotion, cognition, and neuroendocrine interactions. Dopamine acts on five different dopamine receptors (DRs) belonging to the 7-transmembrane G protein-coupled receptor family, which are grouped into two families: the D1-like dopamine receptors D1- and D5DR, which activate adenylate cyclase, and the D2-like dopamine receptors D2-, D3-, and D4-DR, which inhibit adenylate cyclase.^55^ In addition to the regulation of cAMP, several studies have revealed that DR can act through alternative signaling pathways (summarized in ref. ^56^).

Human immune cells express almost all dopamine receptors (DRs) (recently summarized in ref. ^57^). Among leukocytes, B cells and NK cells have the highest DR expression. A previous publication showed that human NK cells express D2-D5DR and lack D1DR.^58^ In general, the activation of DR seems to have an inhibitory function on human NK cells, although the literature on this effect is very limited at present. Upregulation of D5DR in primary human NK cells prestimulated with IL-2 was demonstrated to suppress the proliferation of NK cells and IFNγ synthesis through the NFkB pathway.^59^ The treatment of freshly isolated human NK cells with common serotonin/dopamine receptor antagonists was demonstrated to inhibit NK cell function;^53^ however, this effect may function via the serotoninergic receptors as described above.

Many experiments in animal models have confirmed the modulatory effects of dopamine on the activation of NK cells (Fig. 3). However, there are some contradictory results that could be due to different experimental settings. In a mouse model, D1-like DR stimulation enhanced the cytotoxicity of NK cells from the spleen, and increased D1-like DR expression and cAMP levels, whereas D2-like DR stimulation was responsible for NK cell inhibition.^60^ In contrast, treatment of mice with haloperidol, a D2-like DR antagonist, inhibited NK cell activities.^61^ Paradoxically, bromocriptine, a D2-like agonist, also inhibited NK cell function in the same study, and the combination of both drugs reversed the inhibition. A possible different pathway for the two drugs was suggested to explain the unexpected results (prolactin-dependent or prolactin-independent), but the two drugs also act on other receptors, such as serotoninergic receptors. Interestingly, a study aiming to test the effect of different drugs on mouse NK cells showed an inhibitory effect of only 3 out of 7 tested DR antagonists on NK cells, suggesting that the drugs could also act via other receptors.^62^ In APO-SUS rats, which are highly responsive to dopamine, splenic NK cell activity was much lower than that in hypodopaminergic APO-UNSUS rats, indicating an inhibitory effect of dopamine on NK cells.^63^

Some in vivo stress models also suggested an effect of stress-induced dopamine on NK cell function. Restrained stress in mice resulted in impairment of NK cell cytotoxicity, and this effect could be counteracted by in vivo administration of dopaminergic and adrenergic antagonists prior to stress induction.^64^ Another study demonstrated an increase in cytotoxicity after treatment of male rats with agroclavine, a D1-like DR- and α-adrenergic receptor agonist, while the opposite effect was observed in stressed animals.^65^ It is not clear, however, whether agroclavine predominantly acts on dopaminergic or adrenergic receptors.

A different way to study dopaminergic effects on NK cells is the inactivation of sympathetic or only dopaminergic neurons. The injection of 6OH-DA in rats led to sympathectomy, thus reducing the amount of catecholamines, including dopamine, in the blood. As a consequence, a reduced number of NK cells in the blood and spleen was observed.^66^ The specific ablation of dopaminergic neurons via MPTP treatment led to a decreased immune response and enhanced tumor growth in a mouse model, thus confirming the results above.^67^

Taken together, these results strongly suggest involvement of the dopaminergic pathway in NK cell function. Because NK cells also express other receptors, such as adrenergic and serotoninergic receptors, it is difficult to state specific dopaminergic effects of substances that can bind many of these receptors. Some studies using more specific DR modulators suggest an inhibitory effect of dopamine on NK cells, and dopaminergic modulation as a therapeutic strategy. A recent publication supports the possible clinical relevance of dopaminergic modulation, as the treatment of patients with solid refractory tumors with a small-molecule D2 antagonist in a phase II study led to enhanced NK cell tumor infiltration and induction of cytokines.^68^ Based on these promising results, new studies with specific DR agonists and antagonists are required to better understand how to modulate the dopaminergic pathway in NK cells to achieve therapeutic relevance.

### Epinephrine/Norepinephrine

Epinephrine belongs, together with norepinephrine and dopamine, to the group of catecholamines. Norepinephrine and subsequently epinephrine are synthesized from dopamine. They are found in serum at low concentrations, and can strongly increase during acute stress or exercise. Compared with the more stable glucocorticoids, epinephrine mounts a fast and short stress-response signal.^69^ Both norepinephrine and epinephrine bind to adrenergic receptors, but differ in their activation potencies. Adrenergic receptors belong to the G protein-coupled receptor (GPCR) family and signal through heterotrimeric G proteins. Alpha 2 adrenergic receptor α2-AR signals through Gαi, thereby inhibiting adenylate cyclase and cAMP signals. α1-AR activates the Gαq/11-mediated PLC/IP3/Ca^2+^ pathway.^70^ The beta 2 adrenergic receptor (β2-AR) couples to Gαs and activates the cAMP/PKA/p‑CREB pathway;^70,71^ however, prolonged stimulation of β2-AR can induce switching of G protein specificity toward Gαi, thereby inhibiting cAMP production.^72,73^ In addition, adrenergic receptors can form homo-oligomers and hetero-oligomers with other GPCRs that exhibit distinct G protein specificity (reviewed in ref. ^74^).

Norepinephrine preferentially activates α-AR, while epinephrine is a potent stimulator of β-AR. NK cells express high levels of β2-AR but not β1-AR. In CD16^+^ lymphocytes, the expression of α1- and α2-AR was also detected.^75,76^ The functional effects of epinephrine were mainly attributed to β2-AR (reviewed in refs. ^77,78^); however, epinephrine, but not norepinephrine, was also shown to modulate the expression levels of α1- and α2-AR on NK cells in vivo.^75^

In general, epinephrine and norepinephrine seem to inhibit NK cell cytotoxicity and cytokine production^53,71,79^ (Fig. 3), but treatment with submicromolar concentrations of epinephrine might also enhance NK cell function.^80^ This hypothesis is further supported by the finding that chronic stress through repeated social disruption had a “priming” effect on NK cell function in mice.^81^ Infusion of epinephrine or a physiological increase in epinephrine through stress or exercise increased peripheral NK cell numbers, possibly by inhibition of integrin-mediated adhesion to blood vessels.^82–84^ Interestingly, epinephrine induced the specific relocalization of distinct, highly differentiated NK cell subsets.^85–87^

Stressors can be either acute and short, such as physical trauma or surgery, or chronic and long lasting, such as working in a stressful occupation or providing care for a spouse with severe dementia. Connections between acute stress through trauma or surgery and immune function were already described in the 1980s: NK cell activity in patients undergoing upper abdominal surgery or elective coronary artery bypass grafting was found to be related to the stress response during and after surgery.^88,89^ In addition, decreased NK cell function after traumatic or thermal injury was linked to adrenergic signaling.^90^ Consequently, many clinical studies were conducted in which beta-blockers, usually in combination with COX2 inhibitors, were applied pre- and perioperatively in cancer surgery. Despite the heterogeneity of cancer types and drugs used, the data suggest a beneficial effect of adrenergic receptor blockade on NK cell activity and tumor control (reviewed in ref. ^77^). The extent of NK cell modulation and the resulting diminished tumor control due to surgical stress and β-AR stimulation are affected by age and gender.^91–94^

Chronic stress is known to negatively affect immune function.^95^ Since chronic life stress is difficult to define or control in humans, the majority of studies were conducted in rodents.^96^ Prolonged wet-cage exposure or continuous administration of β2-AR agonists disrupted the immunostimulatory effects of IL-12 on NK cells in rats.^97^ Another study suggested a role for epinephrine in leukemia progression through reduced NK activity in chronically stressed rats.^98^ In addition to tumor control, β2 adrenergic signaling was shown to affect NK cell function against viral infections. Mice treated with a β2-AR agonist showed increased susceptibility to MCMV infection.^99^ Similar findings have been described in humans. For example, daughters of breast cancer patients who experienced high levels of distress exhibited increased concentrations of catecholamines, which were paralleled by decreased NK cell activity.^100^

Moderate physical exercise, psychological interventions, and other stress-reducing techniques were shown to reduce catecholamine levels to counteract the negative effects of chronic stress (reviewed in ref. ^101^). Moreover, mindfulness-based stress reduction (MBSR) techniques increased NK cell activity in healthy volunteers^102^ as well as in breast cancer patients and HIV-infected patients.^103–105^ Therefore, stress-reducing activities lead to lower levels of stress hormones and thus might be beneficial for NK cell function.

Conversely, eustress induced by voluntary wheel running or an enriched environment led to increased NK cell antitumor activity in mice. The authors linked these effects to beta-adrenergic signals, as they could be reversed by the addition of the beta-blocker propranolol.^106,107^ In addition to adrenergic signals, other factors are modulated by enriched environments. The activity of mouse NK cells against glioma was modulated by housing in an enriched environment through upregulation of BDNF and IL-15 in the brain.^108,109^

Interestingly, the lack of β2-AR expression on NK cells impaired NK cell expansion and memory formation in response to MCMV infection, indicating a role of intrinsic β2-AR signaling for optimal NK cell function.^110^ Therefore, the effect of epinephrine and norepinephrine on NK cells is likely dependent on the duration of the exposure, the dose, and the context, which can be influenced by other cytokines and factors.

## Concluding remarks

In addition to the effects of glucocorticoids, serotonin, dopamine, and epinephrine on the NK cell activities described here, several other neurotransmitters and neuroendocrine factors have been shown to influence the activity of these innate immune cells. These molecules include adenosine, acetylcholine, and neuropeptides, and their effects have been reviewed previously.^111–113^ The regulation of NK cells by neuroendocrine factors provides one important mechanistic link between (chronic) stress and changes in NK cell activities. In addition, other factors, such as air pollution, have been shown to result in the release of catecholamines,^114^ which may therefore influence NK cell activities in a similar way. As NK cells are important immune effector cells against cancer, blocking neuroendocrine factors, their receptors, or signaling pathways, may open up novel therapeutic strategies to enhance NK cell functions against tumors. However, not every form of stress seems to have a negative impact on NK cell functions. Acute stress can activate NK cells, and thereby enhance their responses against infections. Therefore, the timing and context of exposure to the different neuroendocrine factors seem to be important. This phenomenon is especially clear during infections. While enhancing NK cell activities during acute infection may help fight the pathogen, limiting NK cell activities is also important to prevent immune-mediated pathologies. This process may explain why neuroendocrine factors were found to have stimulatory and inhibitory effects on NK cells.
